# The Impact of Prior Tonsillitis and Treatment Modality on the Recurrence of Peritonsillar Abscess: A Nationwide Cohort Study

**DOI:** 10.1371/journal.pone.0109887

**Published:** 2014-10-07

**Authors:** Ying-Piao Wang, Mao-Che Wang, Hung-Ching Lin, Pesus Chou

**Affiliations:** 1 Department of Otolaryngology, Head and Neck Surgery, Mackay Memorial Hospital, Taipei, Taiwan; 2 Institute of Public Health and Community Medicine Research Center, National Yang-Ming University, Taipei, Taiwan; 3 Department of Audiology and Speech Language Pathology and School of Medicine, Mackay Medical College, New Taipei City, Taiwan; 4 Department of Otolaryngology, Head and Neck Surgery, Taipei Veterans General Hospital and School of Medicine, National Yang-Ming University, Taipei, Taiwan; Boston University, United States of America

## Abstract

**Background:**

Studies suggest an increased risk of peritonsillar abscess (PTA) recurrence in patients with prior tonsillitis. However, this association is inconsistent and could be confounded by different treatment modalities. This study aimed to assess the risk of recurrence among PTA patients with different degrees of prior tonsillitis and treatment modalities, and the role of tonsillectomy in current practice.

**Methods:**

All in-patients with peritonsillar abscess between January 2001 and December 2009 were identified in a nationwide, retrospective cohort study. Recurrence was defined as the first occurrence of PTA ≧30 days from the initial PTA. Factors independently associated with recurrence were analyzed using Cox proportional hazard model after adjusting for demographic and clinical data.

**Results:**

There were 28,837 patients, with a 5.15% recurrence rate and 4.74 years of follow-up. The recurrence rates were significantly higher among subjects with more than five prior tonsillitis or 1–4 prior tonsillitis compared to those without prior tonsillitis (adjusted hazard ratio, 2.82 [95% confidence interval, 2.39–3.33] and 1.59 [95% CI: 1.38–1.82]). The adjusted HR in patients treated with needle aspiration was 1.08 compared to those treated with incision & drainage (95% CI: 0.85–1.38). After age stratification, the adjusted HRs of more than five prior tonsillitis increased to 2.92 and 3.50 in patients aged ≦18 and 19–29 years respectively. The adjusted HR ofneedle aspiration only increased in patients ≦18 years old (aHR: 1.98 [95% CI: 0.99–3.97]). The overall tonsillectomy rate was 1.48% during our study period.

**Conclusions:**

The risk of PTA recurrence increases with higher degrees of prior tonsillitis in all age groups and management by needle aspiration only in the pediatric population. Patients younger than 30 years old with PTA and more than five prior tonsillitis have the greatest risk of recurrence.

## Introduction

Deep neck infection is one of the most lethal infectious diseases in the complex framework formed by the three layers of the deep cervical fascia, with potential morbidity and mortality ranging from 1.6% to 40% [1]–[4]. Peritonsillar abscess (PTA) is the most common type of deep neck infection, colloquially referred to as “Quinsy”, and accounts for approximately 30% of head and neck abscesses [5]–[7]. Even in the antibiotic era, PTA remains a common condition, with an incidence that has increased by 18% in the United Kingdom over the last 10 years [8], [9]. Appropriate management has been debated for more than two decades. In a National Audit of PTA treatment in the UK, the main strategies are needle aspiration along with antibiotics (60%), incision and drainage (25%), intravenous antibiotics only (5%), and abscess tonsillectomy (1%) [8], [10], [11].

Although all treatments are initially effective, a substantial proportion of PTA tend to recur [12]. The recurrence rate of PTA is poorly defined in the literature, ranging from 5% to 22%, with variability in age, sex, duration of follow-up, and different managements [8], [13]–[19]. To date, there is no direct estimate of the frequency of PTA recurrence in a nationwide study in the world. The higher PTA recurrence rates reported in some observational studies are associated with recurrent tonsillitis prior to the development of PTA [8], [13], [20]–[22]. Conversely, two retrospective studies indicated no significant difference in recurrence rates between patients with and those without prior tonsillitis [14], [19].

The mode of treatment may influence the recurrence rates of PTA. In descriptive studies, patients treated with needle aspiration have a higher incidence of residual and recurrent PTA compared to incision and drainage (I&D) patients [14], [21]. In contrast, a retrospective study of 38 pediatric PTA patients has revealed no difference in different treatment modalities [19]. Data from current literature are inconclusive regarding factors associated with PTA recurrence. Most observational studies are small-sized, unable to analyze recurrent tonsillitis and treatment modalities simultaneously, unable to adjust for important covariates like demographic factors and co-morbidities, have relatively short follow-up periods, and not population-based.

Under the hypothesis that prior tonsillitis and treatment modality are independently associated with increased risk of PTA recurrence, this retrospective nationwide study assessed how recurrent tonsillitis and mode of treatment affected the risk of PTA recurrence. The role of tonsillectomy as PTA treatment remains controversial, as quinsy or interval tonsillectomy is reported in 10–20% of patients after PTA episodes [16], [23]–[25]. This study also provided tonsillectomy data on PTA patients that had not been addressed in a nationwide setting.

## Materials and Methods

### Study design and data source

A retrospective, nationwide cohort study was conducted to determine the recurrence rate of PTA with a specific follow-up period and to investigate the influence of prior tonsillitis and treatment mode on the risk of PTA recurrence. Data were obtained from the National Health Insurance Claims database. The National Health Insurance (NHI) program was launched in March 1995 and covers over 98% residents and medical utilities in Taiwan [26]. The electronic files contained details of health care services for every patient, including demographic characteristics, complete out-patient visits, hospital admissions, diagnoses, prescriptions, clinical orders of participants. The Institutional Review Board of Mackay Memorial Hospital approved this study (12MMHIS129). Because the personal information and identification numbers of the subjects included in this study were de-identified in the Claims database, the review board stated that the written informed consent from patients was not required.

### Study population

All the PTA patients in Taiwan between 2001 to 2009 were identified from the entire population in the NHI program. Claims data from both the out-patient and in-patient database were studied. All subjects with claims data with the in-patient codes relevant to PTA (ICD-9-CM 475) between 2001 to 2009 were enrolled as target subjects. All ambulatory and in-patient claims data, details of in-patient, and ambulatory orders and registry files from 2000 to 2009 were used in the research. The index date was defined as the date of the first PTA episode.

**Figure 1:**
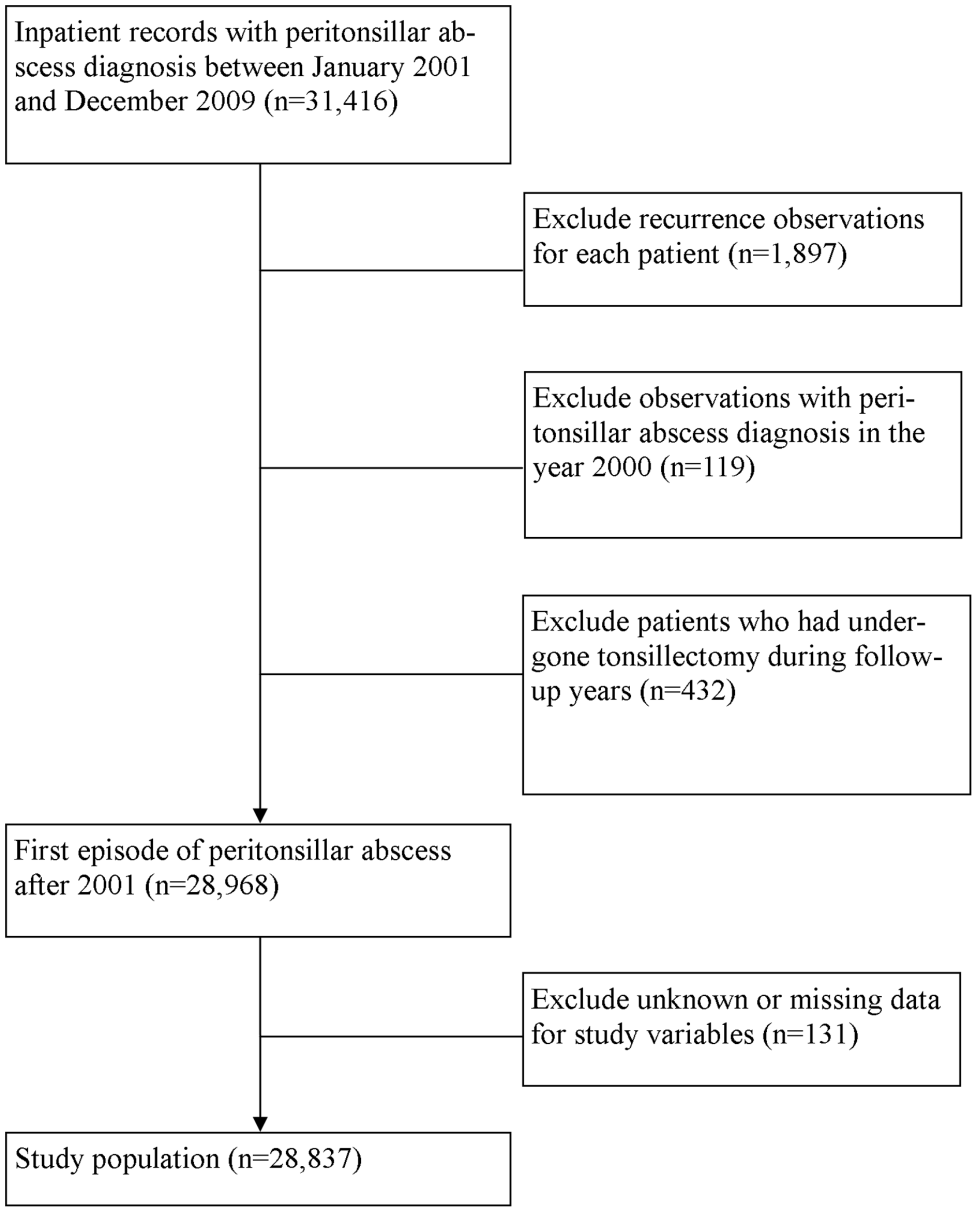
Assembly of PTA cohort.

Patients with abnormal or inconsequent registry data, or missing data; those with tonsillectomy before censoring or PTA occurrence; and those with a PTA episode in the year 2000 were excluded (Fig. 1).

### Outcome definition

The primary study outcome was the first occurrence of any PTA ≧30 days from the index date of the initial PTA [14]. Censor days were determined from the date when the patient had the initial PTA until the date when he or she was defined as having recurrent PTA, death, or the last day he or she was covered by the NHI program for those who did not have recurrent PTA.

### Independent variables

Information regarding demographics, prior tonsillitis, treatment modalities, and co-morbidities were obtained from the claims data of each individual. Age was categorized into three groups for further analysis: ≦18 years, 19–29 and ≧30 years. The patients were also stratified by sex.

To determine a history of prior tonsillitis, the number of out-patient visits for acute tonsillitis in the preceding year before subject’s index date was categorized into mutually exclusive categories: 0, 1–4, and ≧5 visits. The diagnosis of tonsillitis was dependent on the ICD-9 codes in the administrative claims data. Treatment modalities included needle aspiration, incision and drainage, and intravenous antibiotics only. All of the patients included in this study were treated as inpatient and intravenous antibiotics were given regardless of the use of drainage procedures. The overall mean time for inpatient antibiotics were 4.5±4.4 days. The mean time for inpatient antibiotics were 4.4±3.2 days for the patients received needle aspiration, 4.2±2.7 days for incision & drainage and 4.8±5.8 days for antibiotics only.

Ambulatory and in-patient claims data will be searched for the subject’s co-morbidities. Each co-morbidity was defined as positive if the patient had more than three outpatient visits or one hospitalization claim for the specific disease a year before the index date. Co-morbidities include diabetes mellitus (ICD-9-CM codes 250.00–250.90), hypertension (ICD-9-CM codes 401–405), cardiovascular disorder (ICD-9-CM codes 410–414), chronic kidney disease (ICD-9-CM codes 581–583, 585,586), chronic liver disease (ICD-9-CM codes 571), and cancer (ICD-9-CM codes (140–203). Each co-morbidity was analyzed as a binominal variable.

### Statistical analyses

The SAS statistical package version 9.3 was used for all analysis. Cox proportional hazard model was used to estimate the independent association between prior tonsillitis and recurrent PTA after adjustments for numerous confounding factors, including treatment modalities, co-morbidities, and demographic factors. The 95% confidence intervals (CIs) of the adjusted hazard ratios (aHRs) were calculated. Significance was set at a two-sided *p*<0.05.

## Results

### Patient Characteristics

This study included 28,837 patients with initial episode of PTA between 2001 and 2009 in Taiwan. The mean age was 25.5±18.9 years for the entire PTA cohort and 13.8±15.6 years for the recurrent PTA patients. Overall, 19083 (66.2%) were males, 18907 (65.6%) were younger than 30 years old, 20150 (69.9%) had prior tonsillitis, and 14726 (51.1%) were treated with needle aspiration. There were 1486 (5.15%) patients experienced PTA recurrence during a 4.74-year follow-up. The recurrence rate was 6.7% in patients aged <30 years and 2.1% to those aged ≥30 years (*p*<0.0001). The mean time to recurrence was 1.16±1.28 years after initial PTA. Recurrence rates also differed significantly in different degrees of prior tonsillitis, treatment modality and co-morbidities including diabetes, hypertension, cardiovascular disease, renal disease, chronic liver disease and cancer. However, the recurrence rates in females and males did not differ significantly (5.4% and 5.0%, respectively) (Table 1).

**Table 1:** Characteristics of patients with peritonsillar abscess (PTA).

	Peri-tonsillar abscess				
	Total (n = 28,837)	Recurrence (n = 1,486)			
Characteristics	No.(%)	No.	Rate (%)		*p* value
Age, mean (SD)	25.5(18.9)	13.8(15.6)		<0.001	
Age stratification					<0.001
≧30 years	9,930(34.4)	211	2.1		
19–29 years	8,883(30.8)	296	3.3		
≦18 years	10024(34.8)	979	9.8		
Sex					0.12
Female	9,754(33.8)	530	5.4		
Male	19,083(66.2)	956	5.0		
Frequency of tonsillitis (1Y)					<0.001
0 times	8,687(30.1)	258	3.0		
1–4 times	17,324(60.1)	887	5.1		
≧5 times	2,826(9.8)	341	12.1		
Treatment					<0.001
Incision & drainage	2,357(8.2)	76	3.2		
Antibiotics only	11,754(40.8)	530	4.5		
Needle aspiration	14,726(51.1)	880	6.0		
Co-morbidity					
Diabetes	1,005(3.5)	15	1.5		<0.001
Hypertension	1,817(6.3)	35	2.0		<0.001
Cardiovascular disease	766(2.7)	17	2.2		<0.001
Renal disease	272(0.9)	6	2.2		0.03
Chronic liver disease	1,432(5.0)	35	2.4		<0.001
Cancer	259(0.9)	5	1.9		0.03

Abbreviations: SD, standard deviation.

### Prior tonsillitis, treatment modalities and recurrent PTA

Among the 1486 recurrent PTA patients, 82.6% had prior tonsillitis compared to 69.2% in the 27351 non-recurrent cohort (*p*<0.0001). Patients with a history of prior tonsillitis ≥5 visits had a 2.82 fold increased risk of PTA recurrence (aHR: 2.82; 95% CI: 2.39–3.33). The risk of recurrent PTA among patients with 1–4 prior tonsillitis was also significantly different from those without prior tonsillitis (aHR: 1.59; 95% CI: 1.38–1.82) (Table 2).

**Table 2:** Adjusted hazard ratios for the risk of PTA recurrence.

Variable	aHR	95% CI
Age		
≧30 years	1.00	
19–29 years	1.48	1.23–1.79
≦18 years	3.92	3.31–4.64
Sex		
Female	1.00	
Male	1.13	1.01–1.26
Frequency of tonsillitis (1Y)		
0 times	1.00	
1–4 times	1.59	1.38–1.82
≧5 times	2.82	2.39–3.33
Treatment		
Incision & drainage	1.00	
Antibiotics only	0.88	0.69–1.13
Needle aspiration	1.08	0.85–1.38
Co-morbidity		
Diabetes	0.70	0.40–1.21
Hypertension	0.95	0.64–1.43
Cardiovascular disease	1.24	0.72–2.13
Renal disease	1.00	0.44–2.26
Chronic liver disease	1.10	0.77–1.56
Cancer	0.89	0.37–2.17

Abbreviations: aHR, adjusted hazard ratio; CI, confidence interval.

Among 28,837 PTA patients, 14,726 (51.1%) were treated with needle aspiration, 2357 (8.2%) by incision and drainage, and 11,754 (40.8%) by intravenous antibiotics only. The aHR in patients treated with needle aspiration was 1.08 compared to those treated with incision & drainage (95% CI: 0.85–1.38) (Table 2).

### Age stratification

Results of stratified analysis were presented in Table 3. Stratified by age, the risk of PTA recurrence increased significantly in patients aged ≦18 years (aHR: 2.92; 95% CI: 2.37–3.61) and 19 to 29 years (aHR: 3.50; 95% CI: 2.27–5.38) among patients with a prior history of tonsillitis ≧5 visits. The risk of recurrent PTA among patients with a prior history of 1–4 tonsillitis was significantly different from those without prior tonsillitis (aHR: 1.67; 95% CI: 1.37–2.03 in patients aged ≦18 years and aHR: 2.04; 95% CI: 1.54–2.70 in patients aged 19–29 years). In contrast, the impact of prior tonsillitis on the risk of PTA recurrence decreased among patients aged ≧30 years old (aHR: 1.76; 95% CI: 1.02–3.03 for ≥5 prior tonsillitis)and subsequently became statistically insignificant in patients with a prior history of 1–4 tonsillitis (aHR: 1.01; 95% CI: 0.75–1.36). In terms of treatment modalities, the aHR of patients treated with needle aspiration only increased in patients ≦18 years old (aHR: 1.98 [95% CI: 0.99–3.97]); however, no association was found between PTA recurrence and treatment modalities in patients older than 18 years old (Table 3). Overall, the recurrence rate in the high risk group aged under 30 years with ≧5 prior tonsillitis was 13.70% (325/2373) compared to 4.39% (1161/26464) in the others (*p*<0.001).

**Table 3:** Risk of PTA recurrence stratified by age.

	≦18 years of age (n = 10,024)		19–29 years of age (n = 8,883)		≧30 years of age (n = 9,930)	
Variables	aHR	95% CI	aHR	95% CI	aHR	95% CI
Sex						
Female	1.00		1.00		1.00	
Male	1.12	0.99–1.27	1.21	0.91–1.60	1.15	0.86–1.54
Frequency of tonsillitis (1Y)						
0 times	1.00		1.00		1.00	
1–4 times	1.67	1.37–2.03	2.04	1.54–2.70	1.01	0.75–1.36
≧5 times	2.92	2.37–3.61	3.50	2.27–5.38	1.76	1.02–3.03
Treatment						
Incision & drainage	1.00		1.00		1.00	
Antibiotics only	1.54	0.76–3.10	0.89	0.55–1.23	0.93	0.61–1.44
Needle aspiration	1.98	0.99–3.97	0.94	0.66–1.33	0.97	0.57–1.44

Abbreviations: aHR, adjusted hazard ratio; CI, confidence interval.

Note: Adjusted for diabetes, hypertension, cardiovascular disease, renal disease, chronic liver disease, and cancer.

### Tonsillectomy performed in PTA patients in Taiwan

Tonsillectomy patients were included and the data was re-analyzed in terms of tonsillectomy rate. The overall tonsillectomy rate was 1.48% (432/29269) during our study period. In 1486 patients with PTA recurrence, tonsillectomy rate after PTA recurrence was 3.84% (57/1486).

## Discussion

The present study is the first nationwide report of PTA recurrence that includes all PTA patients in the NHI dataset from 2001 to 2009. Overall, the recurrence rate is 5.15% during a 4.74-year follow-up, which is lower than most of the rates reported in other studies [8], [13]–[19]. The reason for this may be that all of the patients with recurrence within one month from the initial episode are considered as residual diseases instead of PTA recurrence and have been excluded from our study. The residual diseases account for up to 40–47% of cases of PTA recurrence in a previous study [14], [27]. The data here also show that 20.5% are residual diseases, accounting for 1.33% of all PTA subjects.

With a large, nationwide cohort, the results here confirm the association that prior tonsillitis increases the risk of PTA recurrence. There is a 2.82-fold relative increase in the risk of recurrent PTA in patients with ≧5 prior tonsillitis during the year preceding the initial abscess compared to patients who did not suffer from prior tonsillitis. The aHR is 1.59-fold higher in patients with 1–4 prior tonsillitis in the preceding year. The risk varied according to the different degrees of prior tonsillitis, and the dose response effect is statistically significant.

Before this study, results have been inconsistent in the literature. Kronenberg conducted a retrospective study and found the patients with previous history of recurrent tonsillitis had four times higher incidence of developing PTA recurrence than those without prior history (40% vs. 9.6%) [20]. However, previous episodes of tonsillitis was not clearly defined by time period and frequency. Savolainen found that patients with more than three episodes of previous tonsillitis had a significantly higher recurrence rate compared to those with three or less episodes of previous tonsillitis. Of note, most of the recurrence (17/19) happened within two months after the initial PTA diagnosis. They might contaminate the residual PTA cases and prevent the establishment of a reliable association between recurrent tonsillitis and PTA recurrence [13]. In contrast, Wolf’s study demonstrated a history of recurrent tonsillitis before PTA did not have a significant increase on the recurrence rate of PTA [14]. A small retrospective cohort study was done in children under 15 years old (n = 38), but did not find any significant difference between the groups with or without a previous history of recurrent tonsillitis [19]. Again, these studies did not define the frequency of recurrent tonsillitis and might have included residual infections instead of PTA recurrence.

The most appropriate treatment of PTA has been debated for more than two decades, with lack of evidence-based studies focusing on the issues of efficacy, patient discomfort, and PTA recurrence [7], [8], [10]. Although most studies indicate that needle aspiration and incision and drainage provide similar efficacy for the initial PTA treatments [7], [8], [10], [12], [28], [29], little evidence has been noted addressing PTA recurrence among different treatment modalities. In a retrospective study with 160 admitted patients, patients treated with needle aspiration are associated with higher PTA recurrence rate compared to those who underwent incision and drainage [14]. The other study revealed no differences in different modes of treatment in 38 children [19]. However, there are some flaws in these studies. First, they used phone interviews and questionnaire to obtain the final recurrence data and might be affected by recall bias. Second, they did not analyze the history of prior tonsillitis simultaneously which was considered an independent risk factor for PTA recurrence. Third, the second study had a very small case number and found no significant difference. This might be a type II error, which represented the case numbers not large enough to detect a significant difference that actually existed. To date, the present study is the most comprehensive evaluation of the treatment modality and prior tonsillitis associated with PTA recurrence accounting for confounding factors such as demographic factors and co-morbidities. The study design here can address some limitations of the previous studies [13], [14], [19]–[21]. Furthermore, the treatment modalities differ greatly between the pediatric and adult populations. In this study, we noted I&D accounted for only 1.55% in the pediatric group, compared to more than 11% in the adult population. Thus, we stratified the PTA cohorts into 3 categories, ≦18 years old, 19–29 years old and ≧30 years old, which was not done in previous studies. The aHR of needle aspiration only increased in the pediatric group (aHR: 1.98 [95% CI: 0.99–3.97]). The possible explainations for this finding may be that I&D was performed only in cooperative child or child under general anesthesia, and it would be easier to drain the abscess completely in this setting than in child underwent needle aspiration. I&D also prolonged the duration of drainage and led to less repeated drainage procedure. On the other hand, repeated drainage procedures are common in PTA patients treated with needle aspiration before the abscess resolved. However, repeated needle aspirations are often difficult to perform in children. Thus, the risk of PTA recurrence increased in pediatric patients treated with needle aspiration compared to I&D patients, but this association was not found in patients aged 19–29 and ≧30 years.

The role of tonsillectomy in the treatment of PTA remains controversial and varies widely among different countries and even within countries [12], [23]. A PTA review mentions that tonsillectomy is indicated in patients with high risk of PTA recurrence, such as a prior history of tonsillitis and <40 years of age [8]. In 2000, 83% of head and neck surgeons in the UK advised interval tonsillectomy in PTA patients with a prior history of tonsillitis [17]. Recent tonsillectomy guidelines for children from the American Academy of Otolaryngology-Head and Neck Surgery (AAO-HNS) suggest that clinicians should assess the need for tonsillectomy in children with recurrent throat infections associated with peritonsillar abscess [24], [25], [30]. In Finland, Wiksten reported the tonsillectomy rate after peritonsillar abscess or peritonsillar cellulitis in patients aged over 6 years. Quinsy or planned interval tonsillectomy was performed on 19.9% (159/798) of patients during a 5-year follow-up period. Delayed tonsillectomy was performed on 25.5% (163/639) of those who were not initially treated with Quinsy or interval tonsillectomy [16]. On the other hand, Mak conducted a small study and observed only 2.9% (2/67) of PTA patients eventually underwent tonsillectomy in a two-year follow-up in the UK [15].

Results of our study shows that the tonsillectomy has been performed in only 432 patients (1.48%) during a 4.74-year follow-up. Furthermore, the tonsillectomy rate after PTA recurrence is 3.84% in 1486 patients with PTA recurrence. These indicate that most of the patients with initial PTA or PTA recurrence in Taiwan are treated successfully with repetition of aspiration or incision and drainage and/or antibiotics. There is no clinical practice guideline existed, and PTA treatment varies widely among different countries. To the best of our knowledge, this is the first study providing nationwide data regarding treatment modalities in PTA patients, including tonsillectomy.

The strength of this study is the use of a nationwide database in Taiwan, which provides all the PTA subjects covered by the national health insurance program (>98%) and enables a comprehensive investigation of the association between prior tonsillitis, treatment modality, and PTA recurrence. The high insurance-covered dataset also minimizes the possibility for selection bias due to loss to follow-up of the PTA subjects. Nonetheless, some limitations of this study should be addressed. First, the diagnoses of PTA, acute tonsillitis, and medical co-morbidities are completely dependent on ICD-9 codes in the administrative database that includes both in-patients and out-patients. Regarding the definition of tonsillitis, there was no clinical data like fever, cervical adenopathy, tonsillar exudate or a positive culture for group A β-hemolytic Streptococcus. Thus, validation of accuracy of diagnoses is not possible by individual medical record review. This is the limitation of administrative data analysis studies. Second, admitted patients with a principal diagnosis of peritonsillar abscess have been chosen to reduce misdiagnosis. However, this may cause selection bias that will limit the application of the findings to all PTA patients. Fortunately, the vast majority (90%) of PTA patients are treated as inpatients in Taiwan, very similar to the report from a national audit in the UK (94% of patients are managed as inpatients) whilst in some other countries such as the USA, outpatient management is preferred for the majority of patients. [11], [23], [28]. Since the majority of the patients are included, this study has produced relatively generalizable results. Overall, given the robust magnitude of the effects with statistical significance in this study, the limitations are unlikely to compromise the results.

In conclusion, this study confirms the associations regarding increased risk of PTA recurrence among patients with a history of prior tonsillitis in all age groups and managed by needle aspiration in pediatric subgroup. Patients younger than 30 years old with more than five prior tonsillitis episodes have the greatest risk of PTA recurrence.
